# Correlation between Taste Threshold Sensitivity and MMP-9, Salivary Secretion, Blood Pressure, and Blood Glucose Levels in Smoking and Nonsmoking Women

**DOI:** 10.1155/2020/4178674

**Published:** 2020-03-12

**Authors:** Sri Tjahajawati, Anggun Rafisa, Nani Murniati, Cucu Zubaedah

**Affiliations:** ^1^Department of Oral Biology, University of Padjadjaran, Bandung 40132, Indonesia; ^2^Department of Dental Health Community, University of Padjadjaran, Bandung 40132, Indonesia

## Abstract

Cigarette smoking can cause taste receptors to increase the taste threshold value. Consequently, the consumption of sugar and salt will not be controlled, therefore causing systemic diseases such as hypertension and diabetes. Nicotine and tobacco in cigarettes can stimulate MMP-9 which plays vital physiological roles in normal tissue growth and repair processes. This study aimed to find the correlation between taste threshold sensitivity and MMP-9, salivary secretion, blood pressure, and blood glucose levels in smoking and nonsmoking women. This was a cross-sectional study consisting of young adult women aged 18–24 years. Subjects were divided into two groups: the nonsmoking and smoking groups. In the combined data of both groups, the sweet taste threshold was correlated with age (*r* = 0.308, *p*=0.008), blood glucose levels (*r* = 0.238, *p*=0.043), and MMP-9 (*r* = –0.297, *p*=0.011). The salt taste threshold was only correlated with systolic blood pressure in the smoking (*r* = 0.440, *p*=0.032) and combined data groups (*r* = 0.260, *p*=0.026). By using partial correlation, it was shown that the relationship between the salt taste threshold and systolic blood pressure was influenced by smoking habits. The sweet taste threshold in women was found to correlate with age, blood glucose levels, and MMP-9 levels. On the other hand, there was a significant relationship between the salt taste threshold in women with systolic blood pressure, which was the only correlation analyzed in sthis study that was found to be influenced by smoking. However, both sweet and salt taste thresholds were not statistically correlated with salivary secretion.

## 1. Introduction

Cigarette smoking can damage the taste threshold through a variety of ways. Notably, hot smoke from burning cigarettes can cause irritation to the oral mucosa continuously, leading to atrophy of the tongue papilla and thickening of the oral mucosa. Simultaneously, this facilitates nicotine to attach and cover the taste receptor cells in taste buds and disrupts the transmission of nerve impulses to the brain, thereby affecting the secretion of saliva so that it can lead to a reduced taste sensation of the tongue [1].

Furthermore, nicotine in cigarettes also affects the size of taste buds. According to an experiment conducted, taste buds on a smoker's tongue were found to be round or flatter than the buds on a nonsmoker's tongue. This change in shape is due to the decrease in the number of taste cells in the taste bud. Along with the reduction in taste cells, which are clustered within the taste buds, there will also be a decrease in taste capacity [2].

Furthermore, excessive consumption of nicotine can damage the mechanism of immunoglobulin A (IgA) synthesis and secretion, leading to low levels of salivary antibodies. In addition, carcinogenic and toxic substances in cigarettes, such as tar, carbon monoxide, aldehyde, phenol, cadmium, and polycyclic aromatic hydrocarbons (PAHs), can cause irritation and inflammation in the salivary glands. Consequently, salivary gland irritation and inflammation cause an increase in salivary secretion at the beginning of cigarette exposure but decreases over time as a long-term effect of smoking. Likewise, it also decreases the secretion of bicarbonate, thus decreasing the salivary pH. All of the above cause damage to the salivary gland cells and tissues, which further cause taste receptors to increase the taste threshold value [3, 4]. Additionally, consumption of sugar and salt can be influenced by the tasting function. Consequently, if there is a decrease in the tasting function, then consumption of sugar and salt will not be controlled, therefore causing systemic diseases such as hypertension and diabetes [5].

Nicotine and tobacco in cigarettes can stimulate matrix metalloproteinases (MMPs), one of which is MMP-9. An increase in MMP-9 is mainly due to the inflammation caused by cigarettes. MMP-9 plays vital physiological roles in normal tissue growth and repair processes, such as neurite growth, embryonic development, blood vessel creation, ovulation, wound healing, and bone formation. However, decreased levels of MMP-9 cause delayed wound healing and insufficient immune response to infection. Almost all common diseases have elevated MMP-9 levels, including a variety of autoimmune diseases, cancer, obesity, diabetes, and heart disease. In saliva, the elevated levels of MMP-9 are evident in patients with oral cancer and periodontitis [6, 7]. However, there has been no study yet on the correlation between taste function deterioration and MMP-9 level in smokers.

This study specifically focused on women since some of the negative effects of smoking may affect women differently in comparison with men due to the differences in anatomy and physiology of both sexes. In regard to taste, previous studies have reported that women have higher sensitivity than men [8, 9]. It may be related to a number of hormonal changes during the menstrual cycle, throughout pregnancy, or during and after menopause that affect the physiology of the entire body including trigeminal function and taste [8].

## 2. Materials and Methods

This was a cross-sectional study. The subject of this study was young adult women aged 18–24 years in Jatinangor, West Java, Indonesia. The age criteria ensured optimal physical condition. Subjects were divided into two groups: the nonsmoking group including those who did not have a habit or history of smoking and the smoking group consisting of those who had a smoking habit of at least 2 years. Subjects who were taking drugs that affected their sense of taste had a history of diabetes and hypertension and suffered from tongue disorders such as hairy tongue and candidiasis, and those who were suffering from flu were excluded.

Data collection was conducted in Padjadjaran University's Integrated Research Laboratory. The data were obtained by measuring the threshold value of sweet and salt taste, salivary secretion (volume and salivary pH), blood pressure values, blood glucose levels, and matrix metalloproteinase (MMP)-9 levels. Permission was obtained from the Ethics Committee of Padjadjaran University (no. 721/UN6.C.10/PN/2017) along with approval from the study subjects through signed informed consent. Data collection procedures were as follows:

1. For the measurement, a sucrose solution and sodium chloride solution were used for the sweet taste threshold and the salt taste threshold, respectively. Concentrations of both the solutions were ranging from 0.005–0.05 M (a minimum to a maximum concentration for taste perception and identification). Subjects were first asked to rinse their mouth with water, and then, the solution was dripped onto their tongue from the lowest concentration until the subject could identify the taste of the solution.
2. To measure the volume, saliva was collected using the spitting method. Concurrently, the pH value was obtained using a pH paper test.
3. A glucometer and test strips were used to assess the blood glucose levels. A mercury sphygmomanometer (@Riester) and a stethoscope with a combination method (palpation and auscultation) were used to measure the blood pressure.
4. The enzyme-linked immunosorbent assay (ELISA) method was used to obtain the level of salivary MMP-9.

Apart from diagnostic procedures, subjects were also asked to fill out a basic characteristic questionnaire including information on identity, duration of smoking, and number of cigarettes smoked per day. The statistical analysis used in this study was Spearman's rank correlation coefficient.

## 3. Results and Discussion

### 3.1. Results

This study included 73 participants, and they were divided into two groups: smoking (*n* = 24) and nonsmoking (*n* = 49).  Table 1 shows that most subjects from the smoking group had the habit for more than 5 years. Moreover, the highest number of cigarettes consumed was 25 cigarettes per day, but that was the case only for one subject, while the majority of subjects smoked ≤10 cigarettes per day (*n* = 13). The average age of the subjects was 22 years. The physiology of the women body in this study is considered homogeneous because it included a group of young adults who were in optimum body condition, so hormonal factors that influenced the state of the body such as menstruation did not become the studied variables.

The data collected were first analyzed based on group division (smoking and nonsmoking); consequently, it was combined to observe the data as a whole. The sweet taste threshold was not correlated to any of the variables in the study when the data were analyzed based on group division. Meanwhile, in the combined data of both groups, the sweet taste threshold was correlated with age (*r* = 0.308, *p*=0.008), blood glucose levels (*r* = 0.238, *p*=0.043), and MMP-9 (*r* = –0.297, *p*=0.011) as shown in Table 2. An increase in the taste threshold indicated a decrease in taste sensitivity. Based on the data analysis from the study, an increase in age and blood glucose levels was associated with a decrease in the sensitivity of taste buds to sweet taste. Meanwhile, there was a decrease in MMP-9 levels along with a decrease in sweet taste sensitivity.

Table 3 shows that the salt taste threshold was only correlated with systolic blood pressure in the smoking (*r* = 0.440, *p*=0.032) and combined data groups (*r* = 0.260, *p*=0.026). This indicates that an increase of the salt taste threshold was associated with an increase of systolic blood pressure in both groups.

By using partial correlation, the data were analyzed again to determine the effect of smoking on the correlation between the various variables in this study. On the one hand, Table 4 shows that smoking has no effect on the relationship between the sweet taste threshold and any of the variables. However, Table 5 shows that the relationship between the salt taste threshold and systolic blood pressure was influenced by smoking habits (*r* = 0.306, *p*=0.009).

## 4. Discussion

Generally, the aging process can alter taste bud morphology and function. The taste buds of older people tend to degenerate more easily, thus causing an increase in the taste threshold. [10] In this study, we found that only the sweet taste threshold was correlated with age. The Beaver Dam Offspring Study (BOSS) in Wisconsin also found the similar result. The study measured the four basic tastes (sweet, salty, bitter, and sour) and also found a statistically significant association between age and sweet taste perception, but not in the three other tastes. The study stated that their subjects were predominantly composed of middle-aged individuals; therefore, their results could not describe what happened in later life. The same reason may be drawn from this study, where the subjects were only young adults (18–24 years); hence, the result of this study could also not represent the overall effect of age on taste sensitivity [8].

The sweet taste threshold in this study was significantly correlated with blood glucose levels. Several studies suggested that the ability to perceive sweet taste influences the blood glucose levels by altering the amount of sugar consumed and dietary and snacking habits. Individuals with lower sensitivity to sweet taste tend to have greater dietary and sugar intake and thus can be at risk of long-term health outcomes, such as obesity, because they need to consume more sugar to have the same taste sensation than those who are more sensitive [11, 12]. Furthermore, previous studies found that there was a significant increase of the mean sweet taste threshold in the diabetic group compared to the normoglycemic group [13, 14]. It is believed that a decrease of sweet taste response is possibly responsible for the deterioration of glycemic control in individuals, since it will elicit insulin release prior to increasing plasma glucose levels when tasting food, commonly known as cephalic phase insulin release (CPIR). In other words, any alteration of sweet taste response will also affect CPIR [15].

The measurement of MMP-9 levels in this study is based on the premise that smoking causes inflammation in the taste bud, which triggers an increase in MMP-9 levels in saliva. However, the results of this study indicate that there is only a correlation between the sweet taste threshold and MMP-9 where smoking is not considered as a factor that affects the correlation through partial correlation analysis. Zinc ion, being one of the organic components of saliva, might be one of the factors that play a vital role in the relationship between the sweet taste threshold and MMP-9. Henkin et al. [16] and Thatcher et al. [17] reported that patients with taste disorder also have a decreased carbonic anhydrase VI (a zinc metalloprotein), which is needed for the activity of many enzymes. The zinc hydrolysis component of the extracellular matrix (ECM) is associated with MMPs that play a central role in normal tissue remodelling, repair, and destruction [18].

This study data showed that a higher salt taste threshold was correlated with a higher systolic blood pressure. Moreover, smoking was found to be the main factor influencing this correlation. Nonetheless, various studies have proven that salt intake is the most significant cause of increase in blood pressure. The alteration of taste sensitivity can therefore affect food consumption. Thus, individuals with a high salt taste threshold tend to add spices to their food. In addition, it is generally known that the nicotine from the cigarette could cause vasoconstriction and hypoxia, thus decreasing taste sensitivity while increasing both systolic and diastolic blood pressure [19, 20]. In this study, only systolic blood pressure was significantly correlated with the salt taste threshold. It was probably due to the reason that the subjects included in this study were only normotensive individuals. In order to obtain more detailed results, the body mass index and stress levels can be studied in subsequent research with a larger sample size by grouping subjects based on several risk factors such as age. However, the results of this study can be used as the basis that systolic blood pressure can be an initial predictor of decreased salt taste sensitivity.

## 5. Conclusions

The sweet taste threshold in women was found to correlate with age, blood glucose levels, and MMP-9 levels. Smoking only affected the relationship between the salt taste threshold and systolic blood pressure, where smoking can cause an increase in the salt taste threshold, thus increasing systolic blood pressure. However, both sweet and salt taste thresholds were not statistically correlated with salivary secretion.

## Figures and Tables

**Table 1:** The characteristics of subjects in the smoking group.

The characteristics of smoking women	Total (*n* = 24)
(1) Smoking (year)	
2	7
3	4
4	1
≥5	12
Average (SD) 4.4 (±2.0)	

(2) Number of cigarettes per day	
≤10	13
≤20	10
≤25	1
Average (SD): 14.8 (±5.4)	

(3) Age (year)	
<20	2
20–24	22
Average (SD): 21.2 (±1.5)	

**Table 2:** Correlation of the sweet taste threshold with age, MMP-9, salivary secretion, and glucose levels.

Correlation of sweet taste threshold	Smoking (*n* = 24)		Nonsmoking (*n* = 49)		Combined (*n* = 73)	
	*r*	*p*	*r*	*p*	*r*	*p*
(1) Age	−0.297	0.158	0.184	0.206	**0.308**	**0.008**
(2) Salivary volume	−0.006	0.977	0.059	0.689	−0.026	0.830
(3) Salivary pH	0.381	0.067	−0.026	0.857	0.062	0.600
(4) Glucose level	−0.235	0.269	0.164	0.261	**0.238**	**0.043**
(5) Duration of smoking	−0.165	0.441	—	—	—	—
(6) Number cigarettes/day	−0.251	0.236	—	—	—	—
(7) MMP-9	0.037	0.865	−0.250	0.083	−**0.297**	**0.011**

**Table 3:** The correlation of the salt taste threshold with age, MMP-9, salivary secretion, and blood pressure.

Correlation of salt taste threshold	Smoking (*n* = 24)		Nonsmoking (*n* = 49)		Combined (*n* = 73)	
	*r*	*p*	*r*	*p*	*r*	*p*
(1) Age	−0.083	0.700	0.233	0.108	0.049	0.683
(2) Salivary volume	0.286	0.176	−0.084	0.568	0.039	0.743
(3) Salivary pH	−0.013	0.951	−0.075	0.607	−0.047	0.694
(4) Systolic	**0.440**	**0.032**	0.246	0.089	**0.260**	**0.026**
(5) Diastolic	0.257	0.226	0.066	0.654	0.104	0.379
(6) Duration of smoking	0.237	0.265	—	—	—	—
(7) Number of cigarette/day	0.039	0.856	—	—	—	—
(8) MMP-9	0.228	0.285	−0.010	0.943	0.094	0.431

**Table 4:** The partial correlation between the sweet taste threshold and various variables when smoking is used as a covariate.

The partial correlation of sweet taste threshold	*r*	*p* value
(1) Age	−0.016	0.896
(2) Salivary volume	0.115	0.336
(3) Salivary pH	0.179	0.132
(4) Glucose level	0.049	0.682
(5) MMP-9	−0.131	0.272

**Table 5:** The partial correlation between the salt taste threshold and various variables when smoking is used as a covariate.

The partial correlation of salt taste threshold	*r*	*p* value
(1) Age	0.085	0.479
(2) Salivary volume	0.017	0.885
(3) Salivary pH	−0.079	0.512
(4) Systolic BP	**0.306**	**0.009**
(5) Diastolic BP	0.171	0.150
(6) MMP-9	0.038	0.753

## Data Availability

The datasets used and/or analysed during the current study are available upon reasonable request.
